# Transcutaneous Spinal Cord Stimulation Enhances Walking Performance and Reduces Spasticity in Individuals with Multiple Sclerosis

**DOI:** 10.3390/brainsci11040472

**Published:** 2021-04-08

**Authors:** Ursula S. Hofstoetter, Brigitta Freundl, Peter Lackner, Heinrich Binder

**Affiliations:** 1 Center for Medical Physics and Biomedical Engineering, Medical University of Vienna, 1090 Vienna, Austria; 2 Neurological Center, Klinik Penzing—Wiener Gesundheitsverbund, 1140 Vienna, Austria; brigitta.freundl@gesundheitsverbund.at (B.F.); peter.lackner@gesundheitsverbund.at (P.L.); heinrich.dr.binder@outlook.com (H.B.)

**Keywords:** gait dysfunction, human, non-invasive, multiple sclerosis, neuromodulation, spasticity, spinal cord stimulation, standing ability, transcutaneous, walking function

## Abstract

Gait dysfunction and spasticity are common debilitating consequences of multiple sclerosis (MS). Improvements of these motor impairments by lumbar transcutaneous spinal cord stimulation (tSCS) have been demonstrated in spinal cord injury. Here, we explored for the first time the motor effects of lumbar tSCS applied at 50 Hz for 30 min in 16 individuals with MS and investigated their temporal persistence post-intervention. We used a comprehensive protocol assessing walking ability, different presentations of spasticity, standing ability, manual dexterity, and trunk control. Walking ability, including walking speed and endurance, was significantly improved for two hours beyond the intervention and returned to baseline after 24 h. Muscle spasms, clonus duration, and exaggerated stretch reflexes were reduced for two hours, and clinically assessed lower-extremity muscle hypertonia remained at improved levels for 24 h post-intervention. Further, postural sway during normal standing with eyes open was decreased for two hours. No changes were detected in manual dexterity and trunk control. Our results suggest that transcutaneous lumbar SCS can serve as a clinically accessible method without known side effects that holds the potential for substantial clinical benefit across the disability spectrum of MS.

## 1. Introduction

The enabling of lost motor function following chronic spinal cord injury (SCI) by epidural spinal cord stimulation (eSCS) has triggered a resurgence of interest in the therapeutic potential of this intervention [1,2,3,4,5]. A parallel advancement in the field was the development of transcutaneous spinal cord stimulation (tSCS), a surface-electrode based neuromodulation method generating currents partially crossing the vertebral canal and affecting spinal segments innervating the lower extremities [6,7,8]. When applied over the lumbar spinal cord, neurophysiological and computer modelling studies have complementarily suggested the activation of large- to medium-diameter afferent fibers within the posterior roots, which are also the principal neural targets of lumbar eSCS for motor effects [6,7,9,10,11,12]. Independent studies have pointed at the potential of tSCS to augment residual voluntary motor function [13,14,15,16] and to decrease lower-extremity spasticity in SCI [17,18,19]. Importantly, the effects on spasticity were shown to last for several hours beyond the application [17,18,19]. Such carry-over effects present a pivotal requirement for a wider use of tSCS, since the surface-electrode based method cannot be applied chronically and the activated neural structures depend on body position [20].

While recent research has put the spotlight on people with traumatic SCI, individuals with multiple sclerosis (MS) were the first and most studied patient cohort in the early investigations of eSCS in motor disorders [21,22]. Attainable improvements included facilitation of standing and walking function as well as amelioration of spasticity—effects that surpassed those achieved by any other intervention available at that time [22,23,24,25,26,27]. Indeed, spasticity is highly prevalent in MS, with those affected facing higher levels of disability in functional domains [28,29]. Another debilitating consequence affecting the vast majority of individuals with MS is impaired walking function [30]. Notably, the early investigations in MS had applied eSCS over thoracic and cervical levels [31,32,33,34], and no study so far has specifically targeted the lumbar spinal cord. Further, tSCS in MS has not yet been investigated.

The objective of the present study was to gain, for the first time, insights into potential motor effects of lumbar tSCS in individuals with MS as well as their persistence after a single intervention. We used a comprehensive set of complementary tests of lower-extremity spasticity, walking and standing ability, and manual dexterity conducted at different time points before and after a 30-min session of 50-Hz tSCS in a statistically sound sample of 16 individuals with MS. Based on previous experience gained in SCI [18,19], we hypothesized that a single intervention would intermediately, i.e., for two hours post-intervention, ameliorate spasticity and associated symptoms and improve walking performance. We further hypothesized that after 24 h, measures would have returned to pre-intervention levels.

## 2. Materials and Methods

### 2.1. Participants

**Figure 1:**
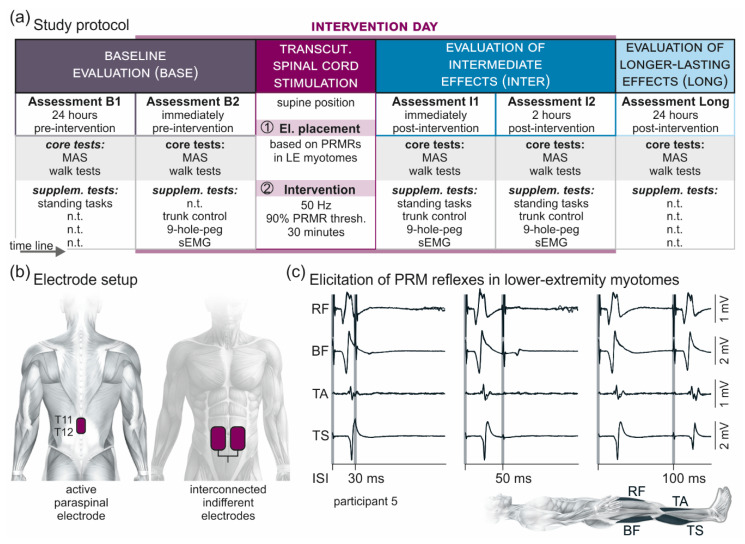
Overview of the methodology. (**a**) Study protocol comprising a baseline evaluation (Base) with two assessments, one conducted ~24 h (B1) and the other one immediately (B2) before a 30-min session of 50-Hz transcutaneous (transcut.) spinal cord stimulation; an evaluation of intermediate carry-over effects (Inter) with two assessments, one conducted immediately (I1) and the other one two hours (I2) post-intervention; and an evaluation of longer-lasting carry-over effects (Long) of the stimulation conducted ~24 h post-intervention. Each assessment included, as core tests, the clinical determination of lower-extremity muscle hypertonia based on the Modified Ashworth Scale (MAS) and, in the ambulatory participants, the timed 10-m walk test, the timed up-and-go test, and the 2-min walk test. Supplementary (supplem.) tests were conducted as indicated in B1 or B2 as well as in I1 and I2, and included standing tasks, the trunk control test, the timed nine-hole-peg test, and a surface-EMG (sEMG) based assessment of lower-extremity spasticity. Transcutaneous spinal cord stimulation was applied with the participants lying supine. First, effective electrode (el.) placement (cf. (b)) over the lumbosacral spinal cord was confirmed based on the elicitation of posterior root-muscle reflexes (PRMRs; cf. (c)) in lower-extremity myotomes. Second, for the intervention, transcutaneous spinal cord stimulation was applied for 30 min at 50 Hz and with an amplitude corresponding to 90% of the lowest PRMR threshold. n.t., not tested. (**b**) Electrode setup with the active paraspinal electrode placed longitudinally over the spine, covering T11 and T12 spinal processes, and a pair of interconnected indifferent electrodes on the lower abdomen. (**c**) Verification of effective stimulation site over the lumbosacral spinal cord by the elicitation of PRMRs in rectus femoris (RF), biceps femoris (BF), tibialis anterior (TA), and triceps surae (TS), which demonstrate a characteristic suppression at interstimulus-intervals (ISIs) of 30 ms and 50 ms when tested by paired pulses [39]. At interstimulus-intervals of 100 ms, the responses to the second stimulus had partially recovered. Shaded backgrounds mark times of stimulus application. Three repetitions each superimposed; participant 5.

Sixteen individuals (mean age ± standard deviation (SD): 56.1 ± 12 years; nine females) with confirmed diagnosis of primary or secondary progressive MS (19.1 ± 13.1 years post initial diagnosis) were enrolled and all completed the study (Table 1). Expanded Disability Status Scale (EDSS) scores ranged from 3.5 to 8.5 [32,35]. Participants 12 to 16 were unable to walk. Participants 4, 7, 9, 12, and 13 were on oral anti-spasticity medication (cf. Table 1) and took the last dose either 12 to 15 h or 36 h (participant 13) prior to the intervention day (Figure 1a). Among the exclusion criteria were acute relapses of MS as well as active and passive implants at T9 or more caudal vertebral levels. None of the participants had any previous experience with tSCS. The study was approved by the Ethics Committee of the City of Vienna, Austria (EK 17-186-1017), and conducted in accordance with the Declaration of Helsinki. All participants signed written informed consent prior to their enrollment into the study.

### 2.2. Study Protocol

We studied intermediate (i.e., lasting for at least two hours post-intervention [19]) and longer-lasting (~24 h post-intervention) carry-over effects of a single 30-min session of tSCS by comprehensive evaluations conducted before and after the stimulation (Figure 1a). The study protocol included a baseline evaluation (Base), comprising two assessments, one conducted ~24 h (B1) and the other one immediately (B2) pre-intervention; an evaluation of intermediately lasting carry-over effects (Inter), comprising two assessments performed immediately (I1) and two hours (I2) post-intervention; and an evaluation of longer-lasting carry-over effects (Long) conducted ~24 h post-intervention. All five assessments (B1, B2, I1, I2, and Long) included the following core tests: the clinical determination of lower-extremity muscle hypertonia based on the Modified Ashworth Scale (MAS) [33]; and, in the ambulatory participants (*n* = 11), the timed 10-m walk test, the timed up-and-go test, and the 2-min walk test. Supplementary tests performed in Base and Inter comprised the assessment of upright standing postural control (conducted in B1, I1, and I2); as well as a surface-electromyography (EMG) based assessment of lower-extremity spasticity [19,34,35], the trunk control test, and the timed nine-hole-peg test (all conducted in B2, I1, and I2).

The MAS-based evaluation of muscle hypertonia comprised flexion, extension, abduction, adduction, as well as internal and external rotation of the hip; flexion and extension of the knee with the hip in an extended position; and ankle dorsiflexion with the hip and knee in a flexed position as well as dorsiflexion, plantar flexion, and pronation of the ankle with the hip and knee in an extended position (twelve separate tests per side) [19]. In each participant, all five assessments were conducted by the same examiner who was otherwise not involved in the study.

For the walk tests, individually required walking aids were unchanged across assessments (cf. Walking Index for Spinal Cord Injury II (WISCI II) Score in Table 1). None of the participants required assistance by physiotherapists. For the timed up-and-go test, participants started sitting on a chair with armrests, stood up on command, walked a distance of three meters, turned around a cone, walked back to the chair and sat down [40,41]. For the timed 10-m walk test [42] and the 2-min walk test [43,44], participants started in a standing position. For the 2-min walk test, participants walked back and forth on a 20-m course, turning around cones at each end.

For the standing tasks, participants were first asked to assume a normal standing position for 30 s, with their bare feet approximately hip width apart, each placed on a separate force plate (type 9284, KISTLER Instrumente AG, Winterthur, Switzerland), and their hands placed to the sides. Subsequently, with both feet placed on a single force plate, mean postural sway was assessed during 30 s of normal stance as well as during Romberg’s test [45], each conducted first with eyes open and then closed. For Romberg’s test, participants stood with their feet close together and their arms straight forward.

The surface-EMG based assessment of different presentations of spasticity was conducted with the participants lying supine and included three repetitions per side of slow, passive unilateral hip and knee flexion-extension movements (3 s each for flexion, holding hip and knee flexed at 90°, and extension), the attempt to elicit an Achilles clonus by a brisk manual ankle dorsiflexion, and cutaneous-input evoked spasms by non-noxious mechanical plantar stimulation with a blunt rod [19]. Care was taken to ensure that participants were relaxed without detectable EMG activity before each repetition of the tests (minimum time between repetitions: 10 s).

Manual dexterity was assessed using the timed nine-hole-peg test [46]. Participants sat at a table and were asked to pick pegs from a container, one-by-one, place them into the holes of a standardized board, then remove them, one by one, and put them back into the container. The test was repeated twice per side.

The trunk control test was used to assess the ability to roll to the (i) weak and (ii) strong side while lying supine; (iii) to sit up from the supine position; and (iv) to maintain balance for 30 s while in a sitting position [47].

### 2.3. Transcutaneous Lumbar Spinal Cord Stimulation

Transcutaneous lumbar SCS was applied through self-adhesive surface electrodes (Schwamedico GmbH, Ehringshausen, Germany), with one electrode (5 × 9 cm) placed longitudinally over the spine covering the T11 and T12 spinal processes and a pair of interconnected electrodes (each 8 × 13 cm) on the lower abdomen, in symmetry with the umbilicus (Figure 1b). A current-controlled stimulator (Stimulette r2x-S1, Dr. Schuhfried Medizintechnik GmbH, Moedling, Austria) was set to deliver charge-balanced, symmetrical, biphasic rectangular pulses of 1 ms width per phase. Such relatively long pulse width was used to exploit the difference in the strength–duration properties between sensory and motor axons and hence to favor afferent stimulation [48,49]. With reference to the abdominal electrodes, the paraspinal electrode acted as the anode for the first and as the cathode for the second phase of the pulse, thereby activating neural structures following the change in polarity of the biphasic pulse [50]. For the intervention, the targeted spinal cord segments were those innervating the lower extremities. Effective stimulation of the respective segmental afferents was neurophysiologically confirmed in the supine position by the elicitation of posterior root-muscle (PRM) reflexes bilaterally in L2 to S2 innervated myotomes, i.e., in rectus femoris (RF), the hamstrings (Ham) muscle group, tibialis anterior (TA), and the triceps surae (TS) muscle group (Figure 1c). Afferent stimulation was verified by testing post-stimulation depression of the evoked responses with double pulses at interstimulus intervals of 30 ms and 50 ms [6,19,50,51]. At interstimulus intervals of 100 ms, the responses typically showed partial recovery [39]. Across participants, the mean PRM-reflex threshold of the first muscle to respond ± SD was 53.9 ± 21.0 mA (per phase of the biphasic stimulation pulse), with a range of 30 mA to 110 mA.

For the intervention, participants remained in the supine position, with additional pillows placed below their knee joints to avoid a completely extended position of the legs, which could exacerbate spasticity [18]. The stimulation mode was changed to the application of tonic stimulation at 50 Hz, a frequency chosen in accordance with previous studies that had used eSCS [52] and tSCS [19] to control spinal spasticity. The stimulation amplitude was slowly increased from 0 mA up to a target intensity of 90% of the PRM-reflex threshold and subsequently applied for 30 min [19].

### 2.4. Electromyographic Recordings

Surface-EMG activity was acquired bilaterally from RF, BF, TA, and TS with pairs of silver-silver chloride electrodes (Intec Medizintechnik GmbH, Klagenfurt, Austria), placed with an inter-electrode distance of 3 cm [34,50]. A common ground electrode was placed over the fibular head of the right leg. Abrasive paste (Nuprep, Weaver and Company, Aurora, CO, USA) was used for skin preparation to reduce EMG electrode resistance below 5 kΩ. EMG signals were recorded using the Phoenix multichannel EMG system (EMS-Handels GmbH, Korneuburg, Austria) set to a gain of 502 over a bandwidth of 10 Hz to 1000 Hz and digitized at 2048 samples per second and channel. EMG data were additionally bandpass-filtered offline between 10 Hz and 1 kHz using a second order Butterworth filter (Matlab 2017b, The MathWorks, Inc., Natick, MA, USA).

### 2.5. Data Analysis and Statistics

Data were analyzed offline using Matlab 2017b (The Math-Works, Inc., Natick, MA, USA) and IBM SPSS Statistics 26.0 (IBM Corporation, Armonk, NY, USA). Assumptions of normality were tested using Shapiro-Wilk test. Datasets were transformed if necessary (log transformations). α-errors of *p* < 0.05 were considered significant. Descriptive statistics of all data were reported as median and interquartile range (IQR).

The MAS-based evaluation of spastic hypertonia resulted in a single score per assessment, the MAS sum score [19]. This was calculated as the sum of the individual MAS scores gained from the twelve different movements tested on each side (with a value of 1.5 for the 1+ scoring category) and could attain a maximum of 96. Individual scores were, 0, no increase in muscle tone; 1, slight increase in muscle tone (catch and release or minimal resistance at the end of the range of motion, ROM); 1+, slight increase in muscle tone (catch and minimal resistance throughout the remaining ROM); 2, marked increase in muscle tone through most of the ROM; 3, considerable increase in muscle tone, passive movement difficult; and 4, affected part rigid in flexion or extension. Changes in individual MAS scores in the evaluations Inter and Long compared to Base were classified as clinically meaningful improvement if reduced by ≥ 1 [19,53,54]; as improvement if reduced by 0.5; unchanged; and increase. For the eleven ambulatory participants, relative improvements of the MAS sum score in Inter compared to Base were additionally calculated and ranked from most (rank 1) to least improvement (rank 11).

The times required to complete the timed 10-m walk test, the timed up-and-go test, as well as the first 20-m course length of the 2-min walk test were measured in each assessment. For the timed 10-m walk test, the walking speed was additionally calculated. For the 2-min walk test, the total distance covered was acquired. Relative improvements of the tests (10-m walk test, timed up-and-go test, time required for the first 20 m and distance covered in the 2-min walk test) in Inter compared to Base were calculated and ranked.

Symmetry of lower limb loading during 30 s of upright standing with each foot on a separate force plate was assessed by obtaining the difference in average load (kg) per limb and normalizing this value to the body weight using the BioWareTM Biomechanical Software Analysis System (type 2812A1-20, KISTLER AG, Winterthur, Switzerland). Postural equilibrium during upright standing on a single force plate was assessed, separately for normal standing and Romberg’s test, each with eyes open and closed, by quantifying the movement of the center of pressure in anterior-posterior and medial-lateral dimensions (sway area) using the BioWareTM Biomechanical Software Analysis System.

Data of the surface-EMG based assessment of spasticity were evaluated by calculating the total activity of the muscles of the examined lower extremity as the sum of the root mean square (RMS) values of the EMG of each muscle, calculated for the passive multi-joint movements from movement onset to offset and for the Achilles clonus and cutaneous-input evoked spasms during 3-s time windows following the onset of the respective stimuli [19]. Additionally, the duration of clonus was measured from onset to the last detectable bout of EMG activity. For each of the three tests, mean EMG-RMS values and clonus durations, respectively, per assessment were obtained by averaging over the three repetitions per leg. For the eleven ambulatory participants, relative improvements of the tests (passive multi-joint movements, Achilles clonus and its duration, and spasms) were additionally calculated and ranked.

The time to complete the nine-hole-peg test was measured and the mean value considering the two repetitions per side (four values) was obtained per assessment.

The trunk control test assigned the following scores to each of the four items tested: 0, if the participant was unable to perform the task; 12, if the participant performed the task, yet with non-muscular help, in an abnormal style, or using the arms for stabilization; and 25, if the participant completed the task without any assistance [55,56]. The total trunk control test score per assessment could range from 0 to 100.

To test for carry-over effects of tSCS on the different outcome measures, separate linear mixed models with time point of evaluation (Base, Inter, and, if tested, Long) as fixed effect, and between subject differences as random effect, were run, thereby Base comprising assessments B1 and B2, and Inter comprising assessments I1 and I2. All post-hoc tests were Bonferroni corrected. Results of B1 and B2 as well as of I1 and I2 were additionally compared using separate Wilcoxon signed-rank tests. Effect sizes were reported by the partial eta-squared (${ŋ}_{p}^{2}$) for linear mixed models or by the correlation coefficient r. For the ambulatory participants, the ranked results of the walk tests were additionally correlated to their WISCI II scores (cf. Table 1) using Spearman’s rank correlation and to the ranked results of the tests assessing different presentations of spasticity (MAS sum score, passive multi-joint movement, Achilles clonus and its duration, and spasms) using Pearson’s correlation, respectively.

## 3. Results

**Figure A1:**
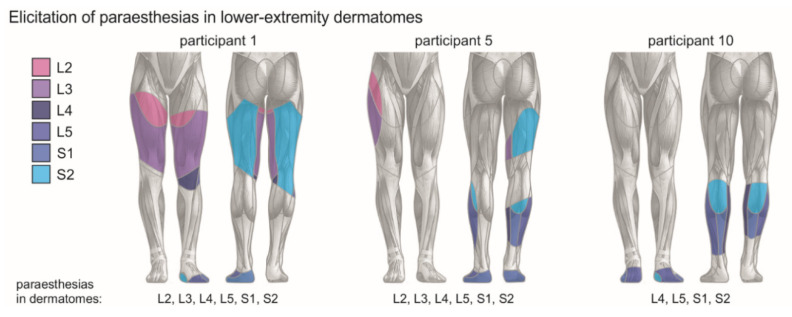
Verification of afferent fiber stimulation during transcutaneous spinal cord stimulation at 50 Hz by the elicitation of paraesthesias in L2 to S2 innervated lower-extremity dermatomes. Exemplary distribution of paraesthesias (shaded areas) as perceived by participants 1, 5, and 10 during the intervention.

Transcutaneous SCS at 50 Hz was applied for 30 min with a mean amplitude of 47.6 ± 18.1 mA, ranging from 27 mA to 100 mA, and corresponding to 88.6% ± 4.6% of the PRM-reflex threshold. The stimulation was well tolerated by all participants. Nine of the 16 participants reported the perception of paraesthesias (tingling sensations) in bilateral L2 to S2 innervated dermatomes during the intervention (Figure A1 in Appendix A) [19]. No discomfort related to the stimulation was reported, nor was there any adverse event. On the intervention day, which lasted ~5 h, the participants reported increasing levels of perceived fatigue (not systematically assessed). Compared to baseline, the intervention produced significant carry-over effects on the individuals’ spasticity, walking function, and, partially, on their standing ability.

### 3.1. Results of Core Tests

#### 3.1.1. MAS-Based Evaluation of Lower-Extremity Muscle Hypertonia

**Figure 2:**
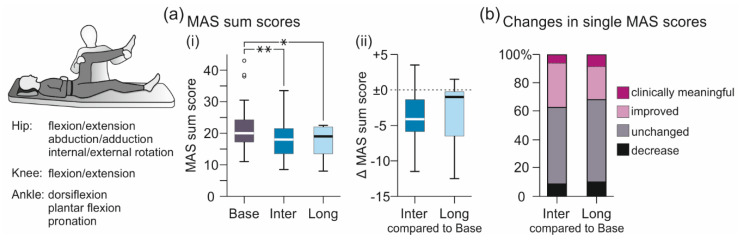
Intermediate and longer-lasting carry-over effects of transcutaneous spinal cord stimulation on the Modified Ashworth Scale (MAS)-based evaluation of lower-extremity muscle hypertonia. (**a**) Group results of (i) MAS sum scores obtained in Base, Inter, and Long; and (ii) changes in MAS sum scores per participant in Inter and Long compared to Base, illustrated by box plots. Bold horizontal lines within boxes are medians; boxes span the interquartile range. Whiskers extend to the lowest and largest values that are not outliers (illustrated as circles; see Methods). Brackets and asterisks denote significant results of post-hoc pairwise comparisons (*, *p* < 0.05; **, *p* < 0.001). (**b**) Changes in individual MAS scores (one score per movement, limb, and participant) in Inter and Long compared to Base. Stacked bar charts show percentage of changes classified as clinically meaningful improvement (reduction by ≥ 1; magenta sections of bars); improvement (reduction by 0.5; light magenta); unchanged (grey); and increase (black). Base, baseline evaluation comprising two assessments conducted ~24 h and immediately pre-intervention; Inter, evaluation of intermediate carry-over effects comprising two assessments immediately and two hours post-intervention; Long, evaluation of longer-lasting carry-over effects conducted ~24 h after the stimulation session; MAS, Modified Ashworth Scale.

Transcutaneous SCS significantly improved lower-extremity muscle hypertonia, quantified based on the MAS sum score both intermediately as well as ~24 h post-intervention (Figure 2a, Table 2). The linear mixed model revealed evaluation (Base, Inter, Long) as significant factor, F(2;54.121) = 14.698, *p* < 0.001, ${ŋ}_{p}^{2}$ = 0.352. Post-hoc comparisons showed a significant decrease of the MAS sum score from Base to both Inter (*p* < 0.001) and Long (*p* = 0.007), with no statistical difference between Inter and Long (*p* = 1.000). Per participant, MAS sum scores in Inter compared to Base decreased by median −4.13 (−6.06 to −1.19) and in Long by −1.00 (−6.87 to 0.13). Of all single MAS scores assessed in Inter, 37.3% were improved compared to Base, with 6.0% rated as clinically meaningful improvement [19,53,54], 54.2% were unchanged, and 8.6% increased (Figure 2b). Of the MAS sum scores obtained in Long, 31.6% were improved, with 8.3% rated as clinically meaningful improvement, 58.2% unchanged, and 10.0% increased.

#### 3.1.2. Walk Tests

**Figure 3:**
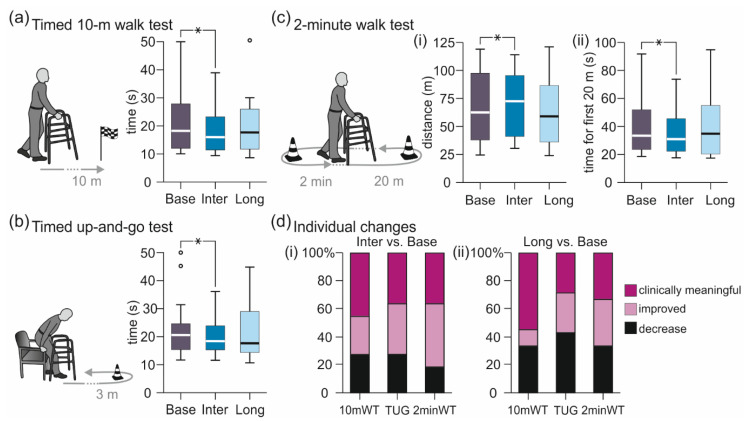
Intermediate and longer-lasting carry-over effects of transcutaneous spinal cord stimulation on walking function. Group results of the evaluations Base, Inter, and Long of (**a**) the timed 10-m walk test; (**b**) the timed up-and-go test; and (**c**) (i) distance of the 2-min walk test as well as (ii) time required to cover the first 20-m course length, illustrated by box plots. Bold horizontal lines within boxes are medians; boxes span the interquartile range. Whiskers extend to the lowest and largest values that are not outliers (illustrated as circles; see Methods). Brackets and asterisks denote significant results of post-hoc pairwise comparisons (*, *p* < 0.05). (**d**) Individual changes in walk tests in (i) Inter and (ii) Long compared to Base. Stacked bar charts of the timed 10-m meter walk test (10mWT), the timed up-and-go test (TUG) and the 2-min walk test (2minWT) show percentage of changes classified as clinically relevant improvement (10mWT, increase of walking speed by ≥ 0.05 m/s; TUG, time reduced by ≥ 15%; and 2minWT, distance increased by ≥ 6.8 m), improvement, and decrease. Base, baseline evaluation comprising two assessments conducted ~24 h and immediately pre-intervention; Inter, evaluation of intermediate carry-over effects comprising two assessments conducted immediately and two hours post-intervention; Long, evaluation of longer-lasting carry-over effects conducted ~24 h post-intervention.

Transcutaneous SCS intermediately decreased the times needed to walk 10 m and 20 m as well as to complete the timed up-and-go test and increased the distance covered in two minutes (Figure 3, Table 2). All group results obtained in Inter, but not Long, were significantly improved compared to Base. The separate linear mixed model analyses revealed evaluation (Base, Inter, Long) as a significant factor; specifically, timed 10-m walk test, F(2;38.055) = 3.671, *p* = 0.035, ${ŋ}_{p}^{2}$ = 0.162; timed up-and-go test, F(2;35.136) = 5.201, *p* = 0.011, ${ŋ}_{p}^{2}$ = 0.228; 2-min walk test, time required to complete the first 20-m course length, F(2;36.059) = 6.894, *p* = 0.003, ${ŋ}_{p}^{2}$ = 0.277; and 2-min walk test, distance covered F(2;38.031) = 3.506, *p* = 0.040, ${ŋ}_{p}^{2}$ = 0.156. Post-hoc comparisons of Base vs. Inter demonstrated significantly reduced times to complete the timed 10-m walk test, *p* = 0.030; the timed up-and-go test, *p* = 0.008; and the first 20-m course length, *p* = 0.003; as well as significantly increased distances covered during the 2-min walk test, *p* = 0.036. None of the Base vs. Long or Inter vs. Long post-hoc comparisons were significant, timed 10-m walk test, Base vs. Long, *p* = 0.821; and Inter vs. Long, *p* = 1.000; timed up-and-go test, *p* = 1.000 and *p* = 0.595; first 20-m course length, *p* = 1.000 and *p* = 0.101; distance covered during the 2-min walk test, *p* = 0.630 and *p* = 1.000.

When comparing the individual results obtained in Inter to those in Base (Figure 3d(i)), 72.8% of the timed 10-m walk tests as well as of the timed up-and-go tests conducted across participants were improved. 45.5% showed clinically relevant improvements in the timed 10-m walk test (walking speed increased by at least 0.05 m/s [57]), and 36.4% in the timed up-and-go test (time required to complete the test decreased by at least 15% [58,59]). Distances covered during the 2-min walk test were increased in 81.8%, with 36.4% showing clinically relevant improvements of at least 6.8 m [60]. Of all participants, the best results in Inter were obtained in participant 9 with an EDSS score of 6.5, who increased her walking speed in the timed 10-m walk test from 0.28 m/s to 0.49 m/s, reduced the time required to complete the timed up-and-go test from 31.4 s to 19.3 s, and increased the distance covered in the 2-min walk test from 31.5 m to 60.5 m.

Across participants and walk tests, the ranked results of the walk tests correlated with the WISCI II scores, Spearman’s ρ = 0.317, *p* = 0.036. Individuals with WISCI II scores of 13 (requiring a walker) and 16 (2 crutches) benefitted the most, while individuals with lower scores of 9 (walker and braces) and 12 (two crutches and braces) or higher scores of 19 (one cane) and 20 (no assistive device) showed less improvement. The ranked results of the walk tests correlated negatively with the ranked results of the tests assessing spasticity (MAS sum score, surface-EMG based evaluation), Pearson’s r = −0.422, *p* = 0.004. Hence, individuals with less improvement in walking performance had a stronger anti-spasticity effect.

When comparing the individual results obtained in Long to those obtained in Base (Figure 3d(ii)), 66.7% of the timed 10-m walk tests were improved, with 55.6% showing a clinically relevant improvement. Of the timed up-and-go tests, 57.1% were improved, with 28.6% of the improvements being clinically relevant. In 66.7% of the 2-min walk tests, distances covered were increased, with 33.3% clinically relevant changes. Of all participants, the best results of the timed 10-m walk test and the 2-min walk test were obtained in participant 4 (EDSS score of 6.0), who increased his walking speed from 0.96 m/s to 1.16 m/s and the distance covered from 104.4 m to 121.0 m, and of the timed up-and-go test in participant 7 (EDSS score of 6.5), who reduced the time required from 23.8 s to 17.6 s.

### 3.2. Results of Supplementary Tests

#### 3.2.1. Standing Tasks

**Figure 4:**
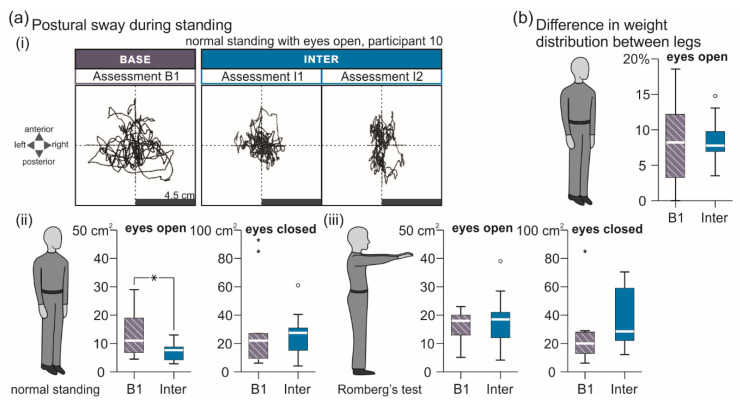
Intermediate carry-over effects of transcutaneous spinal cord stimulation on standing ability. (**a**) (i) Trajectories of the center of pressure in anterior–posterior and medial–lateral directions recorded during 30 s of normal standing with eyes open in B1 and assessments I1 and I2 of Inter; participant 10. Group results in B1 and Inter of postural sway during (ii) normal standing and (iii) Romberg’s test for 30 s with eyes open and eyes closed as indicated. (**b**) Difference in weight distribution (percentage of body weight) between lower extremities during 30 s of normal standing with eyes open shown for B1 and Inter. B1, baseline assessment conducted ~24 h pre-intervention; Base, baseline evaluation; Inter, evaluation of intermediate carry-over effects comprising two assessments conducted immediately (I1) and two hours (I2) post-intervention. All group results are illustrated by box plots. Bold horizontal lines within boxes are medians; boxes span the interquartile range. Whiskers extend to the lowest and largest values that are neither outliers (illustrated as circles) nor extreme values (asterisks). Bracket and asterisk denote significant results of post-hoc pairwise comparisons (*, *p* < 0.05; **, *p* < 0.001).

Transcutaneous SCS intermediately improved balance during normal stance with eyes open compared to B1, while no changes were observed in the other standing tasks (Figure 4, Table 3). Specifically, the area of postural sway (Figure 4a) was significantly reduced in Inter compared to B1 during normal standing with eyes open, F(1;16.146) = 15.700, *p* = 0.001, ${ŋ}_{p}^{2}$ = 0.492, but neither during normal standing with eyes closed, F(1;17) = 0.791, *p* = 0.386, ${ŋ}_{p}^{2}$ = 0.044, nor during Romberg’s test with eyes open, F(1;17) = 0.469, *p* = 0.503, ${ŋ}_{p}^{2}$ = 0.027, or closed, F(1;15.193) = 3.232, *p* = 0.092, ${ŋ}_{p}^{2}$ = 0.175. No difference between B1 and Inter was found for the weight distribution between lower extremities during 30 s of normal standing, F(1;24) = 0.031, *p* = 0.862, ${ŋ}_{p}^{2}$ = 0.001 (Figure 4b).

#### 3.2.2. EMG-Based Assessment of Lower-Extremity Spasticity

**Figure 5:**
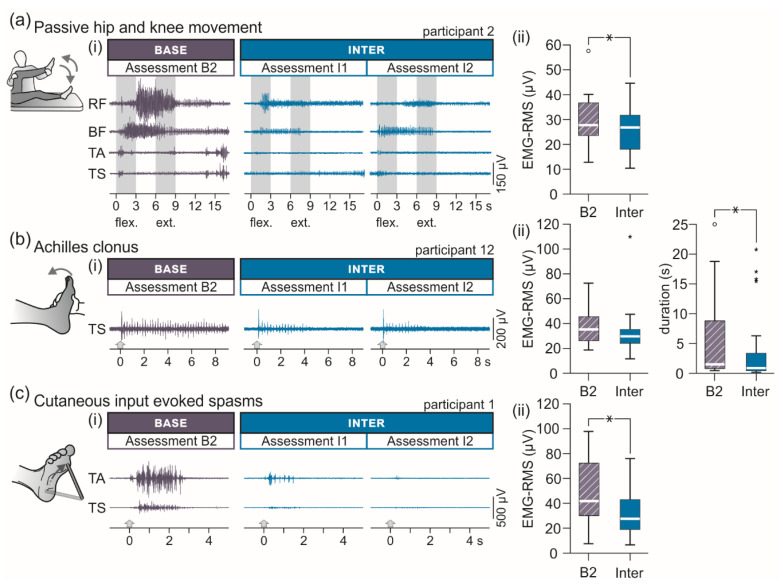
Intermediate carry-over effects of transcutaneous spinal cord stimulation on various presentations of lower-extremity spasticity assessed with surface-electromyography. (**a**) Lower-extremity muscle activation through tonic stretch reflexes during passive unilateral hip and knee flexion (flex.) and extension (ext.) movements. (i) Electromyographic (EMG) recordings derived from rectus femoris (RF), biceps femoris (BF), tibialis anterior (TA), and triceps surae (TS) in assessments B2 as well as I1 and I2; participant 2. (ii) Group results of the root mean square (RMS) values of the EMG in B2 and Inter. (**b**) Achilles clonus elicited by a brisk manual dorsiflexion. (i) EMG recordings of TS in B2 as well as I1 and I2; participant 12. Arrows mark onsets of the tests. (ii) Group results of the EMG-RMS values (left) and clonus duration (right) in B2 and Inter. (**c**) Cutaneous-input evoked lower-extremity spasms. (i) EMG recordings of TA and TS in B2 as well as I1 and I2; participant 1. Arrows mark onsets of the tests. (ii) Group results of the EMG-RMS values in B2 and Inter. B2, baseline assessment conducted immediately pre-intervention; Base, baseline evaluation; Inter, evaluation of intermediate carry-over effects comprising two assessments immediately (I1) and two hours (I2) post-intervention. All group results are illustrated by box plots. Bold horizontal lines within boxes are medians; boxes span the interquartile range. Whiskers extend to the lowest and largest values that are not outliers (illustrated as circles; see Methods) or extreme values (asterisks). Brackets and asterisks denote significant results of post-hoc pairwise comparisons (*, *p* < 0.05).

Transcutaneous SCS significantly reduced lower-extremity muscle hypertonia, spasms, and duration of clonus assessed by means of surface-EMG (Figure 5, Table 3). EMG-RMS values associated with tonic stretch reflexes elicited by passive hip and knee flexion and extension movements, F(1;31) = 10.684, *p* = 0.003, ${ŋ}_{p}^{2}$ = 0.256, as well as associated with spasms induced by non-noxious mechanical stimulation of the plantar surface, F(1;31) = 26.954, *p* < 0.001, ${ŋ}_{p}^{2}$ = 0.465, were significantly decreased from B2 to Inter. In the case of Achilles clonus, no effect on the EMG-RMS was detected, F(1;31) = 3.305, *p* = 0.079, ${ŋ}_{p}^{2}$ = 0.096, yet, clonus duration was significantly reduced, F(1;31) = 11.317, *p* = 0.002, ${ŋ}_{p}^{2}$ = 0.267.

#### 3.2.3. Timed Nine-Hole-Peg Test

No statistical difference was detected between the times required to perform the nine-hole-peg test in B2, 29.3 s (24.4 to 43.1 s), and Inter, 22.4 s (27.9 to 37.3 s), F(1;31) = 1.165, *p* = 0.289, ${ŋ}_{p}^{2}$ = 0.036.

#### 3.2.4. Trunk Control Test

Across participants, scores of the trunk control test remained unchanged from B2, 87.0 (39.3 to 100.0), to Inter, 87.0 (42.5 to 100.0), F(1;31) = 2.067, *p* = 0.161, ${ŋ}_{p}^{2}$ = 0.063. Only two single score changes were observed, both in participant 8, who improved her scores in rolling to the weak and strong sides when lying supine from 12 in B2 to 25 in Inter.

#### 3.2.5. Comparisons between Assessments B1 and B2 and between Assessments I1 and I2

None of the tests performed in B1 or in B2 demonstrated statistical differences (Table A1). Of the 16 tests performed in I1 and I2, the results of two, i.e., of the passive hip-and-knee flexion-extension segment of the EMG-based assessment as well as of Romberg’s test with eyes closed, differed statistically, with the respective values in I1 further improved in I2 (Table A2).

## 4. Discussion

The intervention, 30 min of tSCS at 50 Hz with the targeted intensity, was successfully delivered to all 16 participants. The stimulation reduced lower-extremity muscle hypertonia, clinically determined by the MAS, both intermediately (0 to 2 h) as well as for 24 h beyond its application. The intermediate alleviation was affirmed by the EMG-based evaluation of tonic stretch reflexes during imposed multi-joint movements. Measures of walking ability, including the assessment of walking speed by the 10-m walk test, mobility and fall risk by the timed up-and-go test, and fatigability by the 2-min walk test, were all improved in the intermediate term, but not for 24 h post-intervention. Individuals with greatest improvements in walking performance had comparatively less effect with regard to spasticity alleviation and vice versa. Supplementary tests demonstrated an intermediate decrease in postural sway during normal standing with eyes open, as well as a suppression of muscle spasms, and reduced clonus durations. No changes were found in the upper-extremity nine-hole-peg test nor in the trunk control test.

Transcutaneous lumbar SCS was applied here analogously to previous studies investigating the neuromodulative effects on lower-extremity spasticity in individuals with SCI [17,18,19]. The dorso-ventral electrode montage generates currents through all intervening tissues, with partial current flow across the spine, permitted by the electrical conductivity of its ligaments and intervertebral discs [8,61]. A fraction of the total current is channeled through the vertebral canal, largely through the cerebrospinal fluid in which the spinal roots are immersed, while poorly penetrating the spinal cord [7]. Large- to medium-diameter proprioceptive and cutaneous fibers within the posterior roots have by far the lowest electrical excitation thresholds [11]. The target neural structures of tSCS are hence the same as those largely responsible for the initiation of motor effects by lumbar eSCS [5,6,10,50,62]. The sustained stimulation of lumbar and upper sacral afferent input structures during the intervention was corroborated by the elicitation of paraesthesias in distant dermatomes, here as well as in previous studies [18,19]. Perception of paraesthesias results from the electrical stimulation of A-beta/group II afferents from skin mechanoreceptors [63,64]. Hence, group I afferents with lager axon diameters and lower excitation thresholds must also have been recruited [65,66,67], likely in numbers insufficient to evoke reflex responses in the lower-extremities detectable by surface-EMG [68,69]. Neural activity in spinal and possibly supra-spinal circuits [70] are caused trans-synaptically by the tonic afferent inflow. While the specific circuits recruited remain to be elucidated, they may include last-order inhibitory interneurons [71], interneurons underlying the generation of spasms [72], and those potentiating coordinated motor pattern generation [13,15,73,74,75]. To account for the carry-over effects, the triggered mechanisms must have temporarily altered the transmission through some of the circuits that were sustainedly activated for 30 min [19]. Such persistent effects have been repeatedly reported with stimulation of afferents within peripheral nerves of the lower extremities [76,77,78] as well as with lumbar tSCS in SCI [18,19]. Yet, no study so far has specifically addressed memory mechanisms underlying the temporarily persisting spinal motor effects.

Inflammatory and neurodegenerative demyelination and atrophic processes that can affect white and gray matter at multiple regions of the CNS contribute to the pathology of MS [79]. Among the consequences are spasticity, muscle weakness, and the loss of fine coordination [80]. A primary, life-altering feature of MS that corresponds with the accumulation of CNS damage over time [81,82,83] is impaired walking function, affecting over 90% of individuals within ten years of diagnosis [30]. The walk tests conducted in the present study assessed different aspects of compromised ambulation in MS [84], i.e., walking speed over short distances by the 10-m walk test [85,86], endurance by the 2-min walk test [87], and independent functional mobility by the timed up-and-go test [41]. The intervention significantly improved all walk tests across participants in the intermediate term–despite increasing levels of perceived fatigue during the course of the intervention day. Neurological disability of the eleven ambulatory participants, when classified based on EDSS scores [83,84], was mild in one participant (EDSS score of 3.5), moderate in two (5.0), and severe in eight (6.0 and 6.5). Remarkably, across participants, the best ranked results of all walk tests, both in the evaluation of intermediate and longer-lasting effects, were achieved in individuals with severe disability related to MS. The detected statistical correlation between WISCI II scores [38] and ranked results of the walk tests may hint at the necessity for a critical residual walking ability to be present for the intervention to be effective, while WISCI II scores of 19 and 20 may leave less space for further intermediate improvement [15]. Notably, within the group of eight individuals with severe disability, WISCI II scores showed a wide range from 9 (walker and braces) to 19 (one cane). A priori identification of treatment responders may still remain challenging [70] because of differences in the individual pathophysiology of MS, including affected CNS sites, even with same EDSS and WISCI II scores. Importantly, individuals with the best improvements in walking performance were lower ranked with respect to improvements in spasticity, implying that partially different mechanisms were engaged in the movement-enhancing and the anti-spasticity effects of tSCS.

The intermediate carry-over effects on lower-extremity spasticity in the present study were reminiscent of those previously demonstrated in individuals with SCI [17,18,19]. The complementary tests employed here assessed different presentations of spasticity, i.e., hypertonia, clonus, and muscle spasms that are associated with the altered activity of partially distinct spinal circuits [88]. The exaggerated activation of muscles to externally imposed stretch results from adaptive changes of spinal neural networks and intrinsic properties in spinal motoneurons following chronic lesions to descending spinal pathways. These changes result in a decrease in presynaptic inhibition of Ia terminals [89], reduced homosynaptic depression in the stretch–reflex pathways [90,91], and a reduced strength of post-synaptic inhibition [92,93]. Partially different mechanisms may be involved in the generation of clonus. It is generally assumed that a reduction in motoneuron firing thresholds and re-excitation of stretch reflexes results in the sustained oscillations typically occurring at 5 Hz to 8 Hz [94], while the involvement of a spinal central oscillator was also suggested [95,96]. Contradictory findings exist about the level to which afferent inputs may interfere with clonus [95,96,97]. Spasms are characterized by their preferential elicitation by cutaneous rather than muscle afferent inputs, their long durations, and complex activation patterns across multiple muscles. Animal models of SCI and human studies have suggested an essential role of enhanced activation of persistent inward currents in motoneurons, secondary to the chronic loss of serotonergic innervation, resulting in plateau potentials and self-sustained firing [98,99,100]. Recent studies in mice have demonstrated that the activation of premotor spinal circuits including locomotor-related excitatory interneurons are essentially contributing to the generation of spasms in chronic SCI [72,101]. Given the rich afferent spinal projections, the multi-segmental input generated by tSCS may interact with any of these targets involved in spasticity [19,72,102,103,104].

Early studies using chronic mid-thoracic eSCS in individuals with MS had reported striking effects on upper extremity function, including improved fine hand coordination [21,23]. In a recent study, six weeks of repetitively applied lumbar tSCS in an individual with incomplete cervical SCI improved manual dexterity [19]. Such effects could be mediated via long propriospinal pathways that couple the lumbar spinal cord with the cervical enlargement, or via facilitation of supraspinal circuits by activity in ascending tracts supplied by the stimulated lumbar posterior roots [105,106,107]. We had hence included the timed nine-hole-peg test in our protocol despite the discrepancy between the site of stimulation over the lumbar spinal cord and the spinal segments innervating the upper extremities. A possible explanation for the lack of changes in manual dexterity here could be that such secondary effects would have required repetitive applications of the intervention.

The study protocol comprised two baseline assessments conducted on consecutive days to account for spontaneous variations in spasticity and walking function [108,109]. Across participants, all measures obtained in the two assessments were stable, with no statistical differences detected. In order to minimize the overall strain introduced by the duration of the comprehensive evaluations, not all of the supplementary tests were conducted in both baseline assessments and in the assessment conducted 24 h post-intervention. Yet, for each measure, the comparison between baseline and intermediate conditions was secured. The absence of effects on distant upper extremity motor function suggests that a placebo effect or motivation alone did not play a predominant causative role in the improvements seen in walking performance and lower-extremity spasticity. The lack of changes in the trunk control test could be related to its relatively coarse rating scales, which may have resulted in information loss.

## 5. Conclusions

Given the profound heterogeneity in the pathophysiology of MS, its clinical course, and responsiveness to a particular treatment [110], a critical area for future research will be the prediction of responders to electrical neuromodulation of the spinal cord and to investigate whether attainable effects could be further augmented by personalized stimulation settings [111]. Even before these points will have been ultimately addressed, tSCS, as a non-invasive and clinically accessible method without known side effects that holds the potential for substantial clinical improvement in treatment responders, can be readily applied across the disability spectrum of MS. Responders could further benefit from regular tSCS application over a longer period of time, even in a home-based setting [19].

## Figures and Tables

**Table 1:** Clinical characteristics of study participants.

Part. Nr.	Sex	Age (y)	Time Since Diag. (y)	EDSS Score	WISCI II Score	LEMS Total	PPSS L1 to L2	LTSS L1 to L2	Anti-spasticity Medication (Daily Dosage)
1	F	52	24	3.5	20	38	28	28	None
2	F	66	1	5	20	37	28	28	None
3	M	30	6	5	20	36	25	22	None
4	M	44	7	6	19	39	28	28	100 mg baclofen
5	F	65	24	6	19	31	28	28	None
6	F	71	49	6	19	39	28	28	None
7	F	51	24	6.5	16	34	28	28	75 mg baclofen
8	F	64	29	6.5	16	36	28	28	None
9	F	57	30	6.5	13	43	24	24	62.5 mg baclofen, 12 mg tizanidine
10	M	39	18	6.5	12	38	24	28	None
11	M	61	9	6.5	9	28	28	28	None
12	M	48	3	7.0	0	8	21	28	95 mg baclofen, 300 mg pregabalin
13	F	56	19	7.5	0	19	28	28	10 mg baclofen
14	M	76	20	7.5	0	8	28	28	None
15	F	64	8	8	0	10	14	14	None
16	M	54	35	8.5	0	17	22	22	None

Diag., diagnosis; EDSS, Expanded Disability Status Scale; LEMS, lower extremity motor score (max. 50); LTSS, light touch sensory sub-score (max. 28); Part., participant; PPSS, pin prick sensory sub-score (max. 28); for LEMS, LTSS, and PPSS, see [36]; WISCI, Walking Index for Spinal Cord Injury [37,38]; y, years.

**Table 2:** Group results of core tests.

Test	Base	Inter	Range of Changes Base vs. Inter	Long	Range of Changes Base vs. Long
MAS sum score	20.0 (16.5 to 24.5)	18.0 (13.3 to 21.8)	−11.5 to +3.5	19.0 (12.3 to 22.0)	−12.5 to +1.5
10mWT (s)	18.2 (12.0 to 29.6)	16.0 (11.4 to 23.8)	−14.6 to +1.3	17.7 (10.4 to 28.0)	−9.3 to +1.7
10mWT (m/s)	0.55 (0.34 to 0.84)	0.63 (0.42 to 0.88)	−0.04 to +0.20	0.56 (0.35 to 1.00)	−0.06 to +0.19
TUG (s)	20.6 (14.4 to 25.0)	18.4 (14.9 to 24.2)	−12.1 to +1.5	17.7 (11.2 to 34.0)	−6.3 to +3.8
2minWT, distance (m)	62.5 (37.5 to 98.8)	72.5 (40.9 to 96.6)	−4.8 to +29.0	59.0 (35.6 to 100.8)	−10.0 to +16.6
2minWT, first 20 m (s)	33.7 (21.4 to 55.6)	31.2 (22.0 to 46.8)	−25.1 to +0.7	35.1 (19.6 to 60.1)	−5.7 to +6.7

Base, baseline evaluation comprising two assessments conducted ~24 h and immediately pre-intervention; Inter, evaluation of intermediate carry-over effects comprising two assessments immediately and two hours post-intervention; Long, evaluation of longer-lasting carry-over effects conducted ~24 h post-intervention; MAS, Modified Ashworth Scale; 2minWT, 2-min walk test; 10mWT, 10-m walk test; TUG, timed up-and-go test. Values are medians and interquartile ranges.

**Table 3:** Group results of complementary tests.

**Standing Tasks**	**B1**	**Inter**
Normal stance, eyes open, sway area (cm^2^)	11.0 (5.8 to 19.5)	8.0 (4.3 to 10.4)
Normal stance, eyes closed, sway area (cm^2^)	22.0 (9.5 to 56.0)	22.5 (13.6 to 38.3)
Romberg’s test, eyes open, sway area (cm^2^)	18.0 (11.5 to 20.5)	16.0 (10.6 to 23.8)
Romberg’s test, eyes closed, sway area (cm^2^)	20.0 (12.5 to 28.5)	33.0 (21.0 to 59.0)
Diff. in weight dist. between lower extremities (%)	8.5 (2.5 to 12.8)	9.0 (3.5 to 13.9)
**Surface-EMG Based Evaluation of Spasticity**	**B2**	**Inter**
Passive multi-joint movement, EMG-RMS (µV)	30.5 (23.4 to 37.0)	26.8 (17.9 to 31.9)
Achilles clonus, EMG-RMS (µV)	35.3 (25.9 to 45.9)	29.7 (23.0 to 36.2)
Achilles clonus, duration (s)	1.5 (0.8 to 9.4)	0.9 (0.4 to 3.7)
Muscle spasms, EMG-RMS (µV)	41.8 (29.6 to 73.6)	27.5 (18.8 to 43.8)

B1, baseline assessment conducted ~24 h pre-intervention; B2, baseline assessment conducted immediately pre-intervention; diff., difference; dist., distribution; EMG, electromyography; Inter, evaluation of intermediate carry-over effects comprising two assessments immediately and two hours post-intervention; RMS, root mean square. Values are medians and interquartile ranges.

## Data Availability

The data presented in this study are available in the article.

## Appendix A

**Table A1:** Group results and statistical comparisons of tests conducted in assessments B1 and B2.

Test	B1	B2	Wilcoxon Signed Rank Test
MAS sum score	20.0 (16.0 to 24.5)	20.8 (18.3 to 25.5)	Z = −1.230, *p* = 0.219, r = 0.318
10mWT (s)	19.3 (10.5 to 30.0)	17.2 (12.1 to 31.4)	Z = −0.533, *p* = 0.594, r = 0.178
TUG (s)	20.4 (14.4 to 24.7)	20.6 (14.4 to 30.1)	Z = −1.400, *p* = 0.161, r = 0.495
2minWT, distance (m)	59.0 (33.5 to 98.5)	66.0 (37.0 to 100.0)	Z = −1.599, *p* = 0.110, r = 0.533
2minWT, first 20 m (s)	33.2 (23.5 to 48.4)	33.7 (20.2 to 58.5)	Z = −0.845, *p* = 0.398, r = 0.299

B1, baseline assessment conducted ~24 h pre-intervention; B2, baseline assessment conducted immediately pre-intervention. MAS, Modified Ashworth Scale; 2minWT, 2-min walk test; 10mWT, 10-m walk test; TUG, timed up-and-go test. Values are medians and interquartile ranges.

**Table A2:** Group results and statistical comparisons of tests conducted in assessments I1 and I2.

Test	B1	B2	Wilcoxon Signed Rank Test
MAS sum score	18.3 (13.3 to 23.9)	17.5 (13.3 to 19.4)	Z = −0.910, *p* = 0.363, r = 0.228
10mWT (s)	16.0 (12.7 to 23.3)	16.0 (11.4 to 25.3)	Z = −1.778, *p* = 0.075, r = 0.536
TUG (s)	18.6 (15.3 to 23.9)	18.3 (13.8 to 25.3)	Z = −0.089, *p* = 0.929, r = 0.027
2minWT, distance (m)	73.0 (41.0 to 95.5)	72.0 (40.5 to 100.0)	Z = −0.756, *p* = 0.449, r = 0.228
2minWT, first 20 m (s)	31.0 (22.5 to 43.6)	31.4 (20.5 to 49.8)	Z = −0.663, *p* = 0.508, r = 0.000
Passive multi-joint movement, EMG-RMS (µV)	27.1 (21.0 to 31.9)	23.9 (16.3 to 31.1)	Z = −2.224, *p* = 0.026, r = 0.556
Achilles clonus, EMG-RMS (µV)	30.0 (25.5 to 36.4)	29.7 (16.7 to 36.2)	Z = −1.190, *p* = 0.234, r = 0.298
Achilles clonus, duration (s)	0.9 (0.5 to 4.4)	0.9 (0.4 to 3.7)	Z = −1.138, *p* = 0.255, r = 0.285
Spasms, EMG-RMS (µV)	27.3 (21.6 to 46.9)	31.7 (17.8 to 43.8)	Z = −0.879, *p* = 0.379, r = 0.220
Normal stance, eyes open, postural sway (cm^2^)	8.1 (4.1 to 11.1)	7.3 (4.3 to 10.4)	Z = −0.422, *p* = 0.673, r = 0.149
Normal stance, eyes closed, postural sway (cm^2^)	23.0 (7.2 to 41.0)	22.0 (19.0 to 35.0)	Z = −0.652, *p* = 0.515, r = 0.217
Romberg’s test, eyes open, postural sway (cm^2^)	16.0 (9.5 to 24.5)	16.0 (21.1 to 23.0)	Z = −0.059, *p* = 0.953, r = 0.020
Romberg’s test, eyes closed, postural sway (cm^2^)	33.5 (23.8 to 67.3)	23.0 (15.2 to 54.5)	Z = −2.313, *p* = 0.021, r = 0.818
Normal stance, eyes open, load difference (%)	10.0 (4.5 to 16.6)	8.0 (3.0 to 12.5)	Z = −0.845, *p* = 0.398, r = 0.282
Timed nine-hole-peg test (s)	30.2 (22.6 to 37.3)	27.4 (21.6 to 37.4)	Z = −1.113, *p* = 0.266, r = 0.278
Trunk control test	87.0 (42.5 to 100.0)	87.0 (42.5 to 100.0)	Z = 0.000, *p* = 1.000, r = 0.000

EMG, electromyography; I1, assessment of intermediate carry-over effects conducted immediately post-intervention; I2, assessment of intermediate carry-over effects conducted two hours post-intervention; MAS, Modified Ashworth Scale; RMS, root mean square; 2minWT, 2-min walk test; 10mWT, 10-m walk test; TUG, timed up-and-go test. Values are medians and interquartile ranges.
